# F134. MATERNAL PRENATAL C-REACTIVE PROTEIN AND ADOLESCENT NEURODEVELOPMENTAL OUTCOMES IN THE NORTHERN FINLAND BIRTH COHORT 1986

**DOI:** 10.1093/schbul/sby017.665

**Published:** 2018-04-01

**Authors:** Hugh Ramsay, Golam Khandaker, Heljä-Marja Surcel, Juha Veijola

**Affiliations:** 1 University of Oulu, Royal College of Surgeons in Ireland; 2 University of Cambridge; 3 National Institute for Health and Welfare; 4 University of Oulu, Oulu University Hospital

## Abstract

**Background:**

In utero exposure to infections is associated with adverse neurocognitive outcomes in the offspring. Elevated maternal prenatal serum inflammatory markers, such as C-reactive protein (CRP), have been associated with increased risks of neurodevelopmental disorders, including schizophrenia, later in life. The objective of this study is to investigate the associations between elevated serum concentrations of CRP in early gestation, prospectively assayed in maternal sera, and adolescent psychotic experiences and academic performance. We hypothesised that elevated maternal CRP is associated with adolescent psychotic experiences and poorer academic performance.

**Methods:**

Using data from the Northern Finland Birth Cohort 1986 (NFBC1986), a prospective birth cohort including data since before birth, we examined the association between maternal CRP levels in early gestation (N=7,600) and adolescent psychotic experiences (n=396/5,071) and poorer academic performance (n=2,324/6,770), controlling for sex and maternal education level using multivariable regression analysis. Prior to analyses we determined there was sufficient power to detect small associations (OR>1.68) for these variables.

**Results:**

After controlling for sex and maternal education, those in the highest tertile of prenatal maternal CRP had increased odds of auditory hallucinations (on the PROD-screen) at age 16 years (adjusted OR=1.38, 95% CI: 1.09, 1.74), and poorer school academic performance (beta=-0.08, 95% CI: -0.14, -0.03).

**Discussion:**

Maternal prenatal immune activation is associated with neurodevelopmental outcomes in adolescent offspring. This work extends previous findings regarding prenatal/childhood immune activation and clinical psychiatric diagnoses in adult offspring.

